# Framework for the Analysis of Nanotechnologies’ Impacts and Ethical Acceptability: Basis of an Interdisciplinary Approach to Assessing Novel Technologies

**DOI:** 10.1007/s11948-014-9543-y

**Published:** 2014-04-13

**Authors:** Johane Patenaude, Georges-Auguste Legault, Jacques Beauvais, Louise Bernier, Jean-Pierre Béland, Patrick Boissy, Vanessa Chenel, Charles-Étienne Daniel, Jonathan Genest, Marie-Sol Poirier, Danielle Tapin

**Affiliations:** 1 Department of Surgery, Faculty of Medicine and Health Sciences, Université de Sherbrooke, 3001 Twelfth Avenue North, Sherbrooke, QC J1H 5N4 Canada; 2 Interdisciplinary Institute for Technological Innovation (3IT), Université de Sherbrooke, 3000 Boulevard de l’Université, Sherbrooke, QC J1K 0A5 Canada; 3 Faculty of Law, Université de Sherbrooke, Sherbrooke, QC Canada; 4 Faculty of Engineering, Université de Sherbrooke, Sherbrooke, QC Canada; 5 Faculty of Social Sciences and Humanities, Université du Québec à Chicoutimi, Chicoutimi, QC Canada

**Keywords:** Impact assessment, Nanotechnology, Novel technologies, Risk assessment, Social acceptability

## Abstract

The genetically manipulated organism (GMO) crisis demonstrated that technological development based solely on the law of the marketplace and State protection against serious risks to health and safety is no longer a warrant of ethical acceptability. In the first part of our paper, we critique the implicitly individualist social-acceptance model for State regulation of technology and recommend an interdisciplinary approach for comprehensive analysis of the impacts and ethical acceptability of technologies. In the second part, we present a framework for the analysis of impacts and acceptability, devised—with the goal of supporting the development of specific nanotechnological applications—by a team of researchers from various disciplines. At the conceptual level, this analytic framework is intended to make *explicit* those various operations required in preparing a judgement about the acceptability of technologies that have been *implicit* in the classical analysis of toxicological risk. On a practical level, we present a reflective tool that makes it possible to take into account all the dimensions involved and understand the reasons invoked in determining impacts, assessing them, and arriving at a judgement about acceptability.

## Introduction

It is now widely understood that the ethical questions currently being addressed to nanotechnology have followed in the wake of social reactions to biotechnology’s genetically manipulated organism (GMO) products. True, it would be mistaken to think other technological developments have not been challenged. For instance, the debate around natural versus synthetic chemicals and the use of synthetic chemicals in food packaging (Boseley 2014; Reeser 2013) involves both toxicological questions about food-packaging applications and cultural questions about the status of natural products versus the products of human activity. But the difference between debates like the one over food-packaging chemistry and those surrounding GMOs and nanotechnologies resides in the fact that, with developments in the fields of GMOs and nanotechnology, human activity is transforming nature. About the reaction against GMO products (2011) notes: “Agricultural biotechnology is still reeling from that unexpected revolt, and the proponents of nanotechnology and the still more recent convergent technologies have no wish to see such a marketing debacle repeated” (623). Since then, understanding and lessons about what happened have been sought in the field of biotechnology. The book title *What can nanotechnology learn from biotechnology*? (David and Thompson [Eds.] 2008) clearly illustrates the need for a reflective stance in order to reveal the complexity of the situation and the inadequacies of our way of coping with ethical issues in regulatory science. As one of the book’s editors writes: “What can these antagonists, analysts, and stakeholders learn from the international controversy over the use of biotechnology involving recombinant DNA techniques in agriculture to produce ‘genetically modified organisms’? Biotechnology faced obstacles both in governance (standard-setting and regulatory agencies) and in social acceptance by buyers in the supply chain and by the public” (David 2008, 4).

Numerous issues, and lessons learned from them, have been highlighted in research on the subject, but four of those issues seem to drive ethical research on technological development in general and nanotechnology in particular. The first concerns the questioning of the pact between science and regulatory agencies (Jasanoff 2003). At the core of this issue is the role played by experts and the existing risk-analysis framework in determining social choices. What is risk analysis? What does risk analysis take into account? What is the basis for the decision that a risk is acceptable?

In the USA, for example, the National Nanotechnology Initiative (NNI) clarifies, on its website, the regulatory process involved in risk assessment as follows:

With the advent of new technologies, including nanotechnology, one should consider the potential unintended consequences to human health and the environment that might accompany development and use of the technology. This assessment of the benefits to society and the potential hazards is called risk assessment. The NNI is committed to sound, scientific assessment of nanotechnology benefit and risk, that is, an understanding [of] the potential environmental, health, and safety (EHS) impacts of nanotechnology. (Nano.gov n.d. *Environmental, health, and safety issues*)

As presented in the passage quoted, risk assessment is expert-dependent and seems to be objective. It is this alleged objectivity that we question here.

As Jasanoff (2003) notes: “Claims of objectivity hide the exercise of judgement, so that normative presuppositions are not subjected to general debate. The boundary work that demarcates the space of ‘objective’ policy analysis is carried out by experts, so that the politics of demarcation remains locked away from public review and criticism” (239).

Two components of risk assessment must be distinguished: the scientific component relating to human or environmental toxicity; and the value judgement based on the empirical facts emerging from the scientific component (Grunwald 2005, 191–192). The science at the core of risk assessment is not value-free when it comes to what Godman (2008, 400) calls internal values.

The second of the four issues mentioned above raises the question of what should be considered in the ethical assessment of nanotechnology. If we start with a definition provided by Berne (2004), we can easily grasp the questions that arise about the scope of risk assessment with respect to nanotechnology: “Nanoscaled science and technology, which involves the study, control, manipulation and assembly of multifarious nanoscale components into materials, systems and devices to serve human interests and needs, represents a rapidly developing progression in technological pursuit” (628). The debate on the nature of nanoethics and whether we need a specific field such as nanoethics (Grunwald 2000, 2005; Godman 2008) opposes two views of what should be considered in the ethical assessment of nanotechnologies.

One view argues that the ethical assessment of nanotechnologies must consider only what is specific to nanotechnology, which would mean looking only at the nanoscale science per se and its effects on matter. By definition, nanotechnologies relate to the design, characterisation, production, and uses of structures, devices, and systems by means of control over form and size at a nanometric scale, specifically, between 0.2 nanometres and 100 nanometres (The Royal Society 2004, vii). On a scale of a billionth of a meter—the nanometric scale—matter acquires novel properties that are subject to exploitation. Ethical assessment about nanotechnologies (it is argued) should be limited to these properties.

The other view places greater emphasis on the role played by nanotechnology in social development (Berne 2004; Lekka-Kowalik 2010; Queralto 2013). In this perspective, consideration of the social impacts of technological development as brought about by biotechnology, nanotechnology, or convergent technologies cannot be limited to one technology on its own: each technology intensifies the social impacts that must be reconsidered. Furthermore, we must keep in mind that, as we saw in the passage quoted from the NNI website, risk assessment rests not only on risk analysis but also on social benefits. Usually, these social benefits are measured by the benefits yielded by nanoproducts. Thus one way or another, the assessment of social benefits is required in risk assessment.

The third of the four issues is that of ethical assessment itself. Once we recognize the value-laden nature of risk assessment and the value weighting assigned to social benefits in devising regulations, the question that cries out for an answer is how to carry out an ethical assessment of a nanotechnology. Studies on moral and ethical assessments published in the journal *NanoEthics* showed that these assessments usually end in a deadlock (Patenaude et al. 2011; Béland et al. 2011). We have sketched a way out of this stalemate position (Legault et al. 2013) by adopting a pragmatic view of ethics based on values. Queralto (2013) too embraces the option of a pragmatic ethics in the ethical assessment of nanotechnology: “[P]ragmatic values provide ethical solutions by means of another type of structure, namely, a dynamic and adaptive structure according to the quality of problems to be considered. Hence, the pragmatic ethical view suggests that we change from an axiological pyramid to a certain flexible system of values, a dynamic and adaptive axiological system” (19).

But if ethical assessment rests on values, how can this be made operational? Murphy and Gardoni (2008) have presented a capabilities-based approach to determining the tolerability and acceptability of societal risks. They set out a necessary condition for any framework of acceptability, one of clearly identifying how value judgements are framed: “An approach to acceptable risk evaluates the likely societal impact of a hazard. Such evaluations should be made on the basis of criteria that specify, or are based on, judgements regarding values that ought to be protected, promoted, and prioritized. An approach should offer an explanation of why a particular value or set of values is important and a principled basis for prioritizing competing values in a given way” (79–80).

Finally, the fourth issue relates to the democratic inadequacies of the existing regulatory approach to technological development. France’s experiment in public dialogue on nanotechnology is instructive. From 15 October 2009 to 24 February 2010, a public debate on nanotechnology was held in France (Commission nationale du débat public 2009). The process was an attempt to bring into public debate the complex question of technological development. This experiment in democracy highlighted the fact that participants did all not share the view that only the environment, health, and safety (“EHS”, as abbreviated in the NNI passage quoted above) should be considered; they found this too limited to determine social acceptability.

Ethical reports on nanotechnology and GMOs such as those produced by the Commission de l’éthique de la science et de la technologie du Québec (CEST) tend to reach the same conclusion. The critique voiced addresses the limits of considering only the toxicological impacts of given products and not the impacts of product uses, still less the impact of the techno-science that a product is part of (see CEST 2004/2006). Finally, Jasanoff (2003, 2005, 2011) explores the gap between current regulatory practices and democratic requirements for accepting a technology.

In light of the preceding, we can isolate a focal point for the four issues: the challenge of developing an interdisciplinary framework for the analysis and acceptability of the impacts of nanotechnology. Redefining the pact between science and regulation demands an interdisciplinary approach under which the humanities and social sciences are wedded to the natural sciences in the performance of an impact analysis. Various reports have noted the need to fill the communications gap between disciplines: “Gaps in communication between different scientific disciplines—from the natural, technical and environmental sciences to the economic, social and psychological disciplines—limit the ability to fully consider and act on potential innovations and risks” (IRGC 2007, 14). If we want to discuss the assumptions each discipline makes about the matter, the challenge posed by the communications gap must be met at the very start of the process of creating a practical framework. A comprehensive approach requires discussion about the scientific measurement of risk; a critical view of the values underlying legislation and regulations; and a psychological, sociological, and philosophical discussion of “social” or “societal concerns” (IRGC 2007, 8, 18, 22, 23, 26) and their role in risk evaluation. Such an approach, leaving behind the limited scope of an EHS-based examination of toxicological issues, would aim for comprehensiveness and therefore include an examination of all the impacts on society of a technology’s uses. In order to develop and undertake such an approach, the place of ethics and the role of ethics in evaluating democratically responsible technological development must be clearly stated. The present article proposes a framework for doing just that. The framework in question integrates components of the four issues at stake as laid out above.

In Part 1, we discuss the many uses of the concept of acceptability in the regulation of technological development. In line with Murphy’s and Gardoni’s (2008) requirement for a framework, we clarify the meaning of ethical acceptability.

In Part 2, we present an interdisciplinary frame of reference for analysing impacts and acceptability that, in our view, allows for an integration of the approaches of the natural sciences and the social sciences and humanities that will further a more comprehensive understanding of the issues associated with technological development. This presentation is illustrated with a case that has been analysed as a part of our research, the case of a polymer shoe sole that integrates a pressure sensor designed to enhance diabetic care.

## From the Social Acceptance of Risks to the Ethical Acceptance of Impacts

### The Social Acceptance of Risks

It is said that the Chinese ideogram for “crisis” incorporates the ideas of danger and opportunity; that is, it evokes confronting a critical moment when change is possible. From that perspective, crisis is an appropriate name for the response to agricultural biotechnology’s GMO products. According to the analysis provided by Berne (2004), at stake are how we deal with the social acceptance of technology and the shift to a new way of conceptualizing responsible technological development:

Technology tends to emerge from unreflective social acceptance and passivity as fueled by the influences of free market economies, competition, and perceived needs for more and new material possessions. Commonly, members of these societies become eager for the elusive but compelling promise of improved quality of life. The widespread tendency is to be unconsciously driven by, changed by, and given over to novel technological development. However, technological development is not an inevitable process of evolution for the human species. Rather, it is a choice. Its direction can be willed and determined by conscientious focus on that which is believed and understood about humanity and its relationship to the technologies that are developed. (635)

From a sociological point of view, socialisation creates ways of thinking and evaluating that are embedded in social practices. In this light, a closer look at the social acceptance model of technology starts with the pact between science and regulation in our societies (Jasanoff 2003).

Let us return to the thinking exemplified by the NNI. In the website passage previously cited, we encountered the idea of EHS issues. Here is a passage from another page of the NNI’s website:

An important component of responsible development is the consideration of the ethical, legal, and societal implications of nanotechnology. How nanotechnology research and applications are introduced into society; how transparent decisions are; how sensitive and responsive policies are to the needs and perceptions of the full range of stakeholders; and how ethical, legal, and social issues are addressed will determine public trust and the future of innovation driven by nanotechnology. (Nano.gov n.d. *Ethical, legal, and societal issues*)

This is a perfect illustration of the social acceptance model taken for granted in North American democracies. EHS issues sit, as seen earlier, at the core of the scientific risk assessment. Ethical, legal, and social (ELS) issues are viewed as important but are not involved in risk assessment. On what assumptions is this distinction based?

The first assumption relates to the regulatory process itself and to its role in democratic societies. Broadly speaking, legal authorities enact regulations as a mode of intervention in social practices. The regulatory process can ban certain practices, such as human cloning; set conditions for the use of certain products, such as dangerous materials; or not regulate at all. Since regulation implies the use of social force to attain its aim, there must be a justification for intervention. Any state regulation is a limit to the free market, and the EHS/ELS distinction is consistent with the priority given to the free market over other values. Technological development is free-market driven: the constant search for a “killer app”, that is, of a powerfully marketable use, is the principal aim of the creation of technological products. This is why researchers in the fields of nanotechnology, like researchers in biotechnology before them, declare without hesitation that these technologies will save the world by solving all its main problems (Gordijn 2005). In this perspective, State action can only be justified if and only if harm to the environment, health, and safety is so great that it tilts the scales when weighed against economic gain. This is what currently constitutes “risk assessment”. Murphy and Gardoni (2008) detail the cost/benefit approach taken by the regulatory process as follows:

The most common form of formal analysis is cost-benefit analysis in which the unit of measure is monetary. This approach adds up the risks and benefits of various courses of action. It measures risks in terms of the amount of money people are willing to pay to avoid, or the compensation they would demand for exposure to, certain risks. Thus, it defines risks and benefits in terms of individuals’ subjective preferences, as reflected in market behavior. (83)

The core value judgement in risk analysis is monetary and social acceptance is measured by how much people are willing to pay.

As MacPherson has pointed out (2008), judgements about safety are different than risk-acceptance judgements, since the latter involve not just judgement on the nature of risks but also a weighting of risks and benefits. Because weighting is included in risk analysis, the focus will be on finding risks that are so significant that they outweigh monetary benefits. For questions of human health, lethal risks will be given consideration; for questions relating to the environment, “serious and irreversible damage” (to use the wording of the Rio Declaration; UNEP 1995).

Research conducted within the social sciences on ELS issues and related to social acceptance follows the same embedded assumption, that of an economic model under which individuals are believed to weigh their own losses and benefits when accepting a technological product. In free-market technological development, the consumer’s acceptance is a guide to the measurement of social acceptance. The issues that drive research focus on risk perception and the role it plays in acceptance. Under the logic of individual acceptance of products carrying a risk, and in the context of a society that only protects against serious risk, the term *risk* relates to the danger of death or injury (Sjöberg 1998; Slovic et al. 1979).

Historically, the study of risk perception developed along two major lines, one associated with psychosocial approaches and the other with cultural approaches. The first, which emerged from a psychometric paradigm, aimed to identify individual characteristics in the face of manifest risks (Fischhoff et al. 1978); cultural theory, which may be considered sociological, aimed to identify group characteristics (Marris et al. 1996; Jernelov and Svedin 1998). The goal of studies of these kinds was to establish predictive factors for behaviours. It is easy to guess that studies such as these, conducted in a behaviourist framework, were used for the purpose of winning public opinion over to the acceptance of products; in other words, for marketing purposes. This analytic model, deployed during the seventies, satisfied the needs of that period; namely, to ensure, by means of appropriate marketing strategies, that products emerging from technological development met with acceptance.

The requirement embedded in risk analysis that lethal, serious, or irreversible damage must outweigh monetary benefits if regulatory action is to be taken explains why debate on nanoethics is in a state of deadlock in which, in Gordijn’s terms, utopian dreams stand opposed to apocalyptic nightmares (Gordijn 2005). Certain studies in the humanities and social sciences view risk perception as an apocalyptic nightmare and accordingly analyse fear. In the mainstream scientific view, risk analysis is a scientific endeavour and fear is considered to be an irrational way of measuring risk. Studies on the perception of technological risk have tried to quantify the gap between the true state of scientific knowledge and the state of knowledge by the general public (Sjöberg 1998). These studies have made it possible to recognise and critique the prevailing view of the public understanding of science, termed the “deficit model of public understanding” (Gregory and Miller 2000). Under the deficit model of public understanding, experts are seen as issuing objective judgements about a given risk while members of the public are seen as having a subjective, often emotive judgement. To overcome purportedly irrational fears, it is supposed to be sufficient to provide members of the public with enough information for them to arrive at an objective judgement. Predictably, the scientific communication of the nature of risks is perceived as the answer. Here again the familiar assumption is at work: risk acceptance is objective and does not involve value judgements.

When research in the humanities and social sciences is grounded on that same assumption, its role is to promote social acceptance based on an ELS perspective. As illustrated in Terrade et al. (2009), this expectation persists as a prevailing view of the uses of the social sciences:

[As France’s] Minister for Research has said, “Mastery of uses is a major issue for the economy and society: technologies will only serve as the engines of sustainable economic development if the use that is made of them is observed and taken into account. Understanding the conditions for society to take ownership of technologies has become an essential factor in competitiveness.” It is therefore crucial to have available several models with which to answer these two questions:

(1) What is it that results in our using a novel technology or a novel procedure?

(2) How can we predict the use that will be made of a novel technology that is placed at the disposal of users? (384)

The International Risk Governance Council’s policy brief *Nanotechnology Risk Governance* (IRGC 2007) tries to address the inadequacies of classic risk governance when applied to nanotechnology. The policy brief’s authors write, “Because nanotechnology raises issues that are more complex and far-reaching than many other innovations, the current approach to managing the introduction of new technologies is not up to the challenge posed by nanotechnology. Decision makers worldwide need to work towards a system of risk governance for nanotechnology that is global, coordinated, and involves the participation of all stakeholders, including civil society” (IRGC 2007, 4). The policy brief is targeted towards policy makers, and it therefore reproduces classical risk governance assumptions while trying to adapt that approach to the complexity of nanotechnology. Its stance remains rooted in the legal tradition, in which legal intervention cannot be justified unless the population is subject to serious risks. The policy brief’s proposed framework (IRGC 2007, 23) thus reproduces the traditional distinction between EHS issues and ELS issues, despite the opening made to the concept of “concern assessment”. In this framework, Risk Appraisal requires Risk Assessment, which reproduces the traditional toxicological analysis (Hazard Identification & Estimation; Exposure & Vulnerability Assessment; Risk Estimation). It also requires Concern Assessment, which integrates Risk Perception, Social Concerns and Socio-Economic Impacts. But the policy brief does not make clear what socio-economic impacts are to be considered or how they are to be documented. In traditional risk governance, the final judgement as to the acceptability of risks rests on a cost/benefit calculation, and risk perceptions and social concerns are likely to be addressed by finding methods of communication that will maximise social acceptance.

### Ethical Acceptability

In our introductory discussion of the GMO crisis, we focussed on four issues for which the assumptions of an EHS/ELS-based regulatory model can be challenged and the frame of reference of ethical acceptability can be advanced as an alternative to that of social acceptance. Recourse to the principle of ethical acceptability is also grounded in assumptions.

The first of these concerns the social regulation of technological development. As we have seen, in a free-market perspective, technological development is viewed as a social phenomenon that can be accelerated by research on whatever social forces are involved. On this view, from an ethical perspective nobody is responsible. With a title that recalls an old joke to make a serious point, Davis (2012) summarizes the assumption made about ethical acceptability: “‘Ain’t no one here but us social forces’: Constructing the professional responsibility of engineers” (13). The argument that social forces are at work governing the free market conveys the idea that no one is responsible for technological development, not even the States that finance it at great expense. It is worth repeating a portion of the passage from Berne (2004) quoted above: “[T]echnological development is not an inevitable process of evolution for the human species. Rather, it is a choice. Its direction can be willed and determined by conscientious focus on that which is believed and understood about humanity and its relationship to the technologies that are developed” (635).

In an ethical, moral, and indeed legal perspective, responsibility implies choice. In technological development, there are individual choices, institutional choices, and social choices (Pidgeon et al. 2011; Senjen and Hansen 2011).

With its focus on choice as opposed to reaction to fears, the principle of ethical acceptability is framed in terms of value judgement and complex value judgement. Of course, there are many ways of defining value judgements and of making them operational in the decision-making process. In the model developed in the next section we consider, as argued by Legault (1999), that value judgements constitute one of three decision-making stages. Accepting a technological product by buying it or using it is an action consequent on a decision-making process. Deciding is often spontaneous and not reflective, but when we take a reflective stance, we can see that responsible decision-making involves four steps. The first step consists of the analysis of the possible consequences of the action if we undertake it. The second consists of an evaluation (a value judgement made about the consequences), the third of weighting the conflicting value judgements, and the fourth of giving stakeholders a response justifying the decision taken.

Models for determining ethical acceptability can be used in instrumental, interpretative, or normative ways (Jasanoff 2011, 624). Murphy’s and Cardoni’s capacity-based model (2008, 77) offers a normative alternative to the traditional model of risk acceptability. Our model is essentially interpretative: it aims at an understanding of the diversity of assessments of technological development, what is specific to each, and why they are compatible. It was developed by an interdisciplinary research group working on an interdisciplinary model of impact analysis and ethical acceptability. It is grounded in an analysis of the literature on nanotechnology in which multiple national and international reports were compared with a view to understanding the current deadlock in the field of moral philosophy about nanoethics and the ethics of technology. It keeps constantly in view the epistemic presuppositions of the natural sciences, the humanities, and the social sciences.

## A comprehensive Analysis of Impacts and Acceptability

What are we to understand by a comprehensive analysis of impacts and acceptability? This type of analysis seeks essentially to complement traditional risk analysis. It does so on one hand by taking account of impacts besides those to individual health and safety. On the other hand, it postulates that a decision about acceptability rests not only on risks to be avoided but also on a weighting of the negative and positive impacts for certain social issues.

**Fig. 1:**
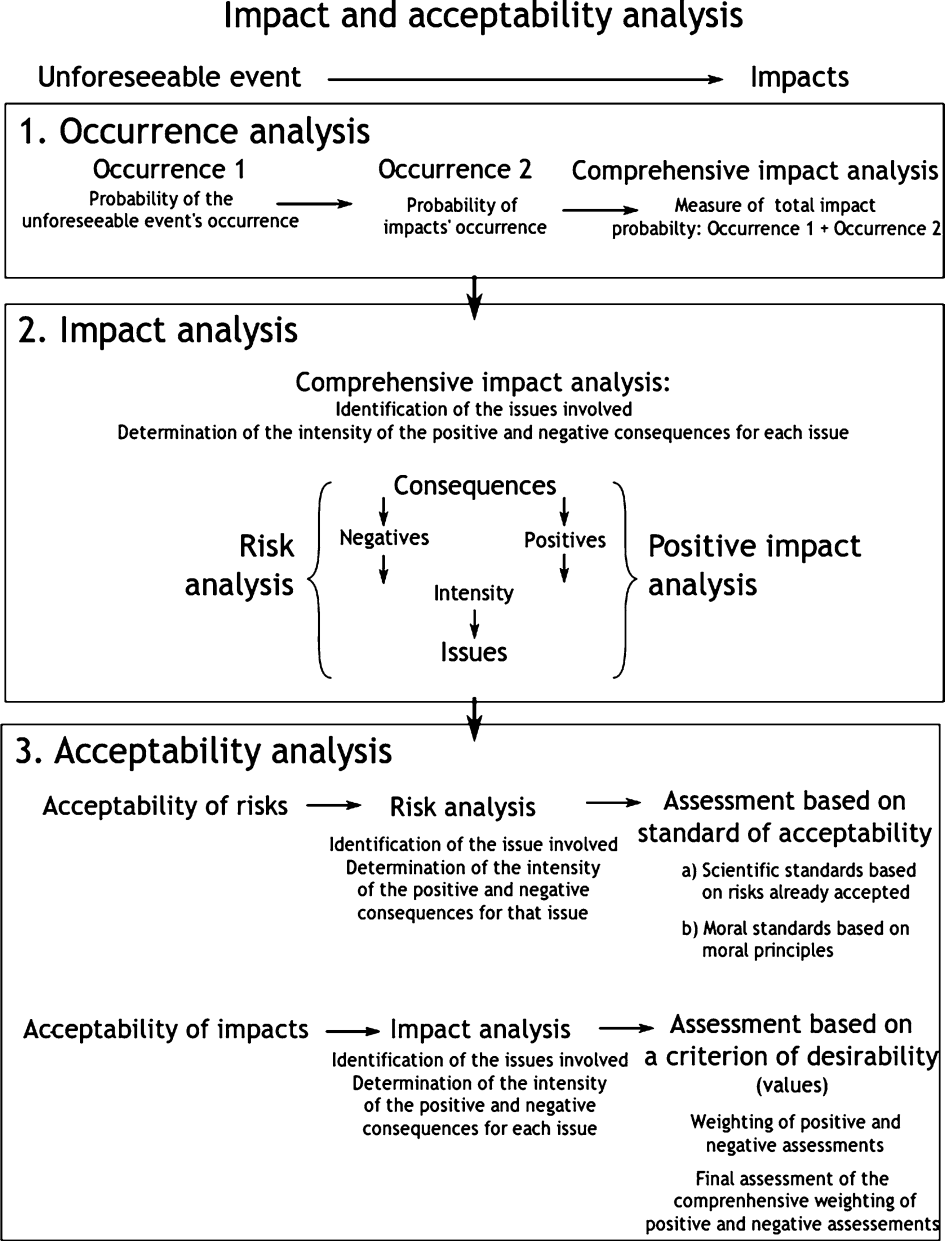
Theoretical frame of reference for the analysis of impacts and their acceptability

The figure below presents the theoretical frame of reference for this framework for analysis. This frame of reference translates in practice into a dynamic process of analysis of impacts and acceptability. Section “Presentation of the process of impact and acceptability analysis devised by our team” below presents a systematic exposition of the process (Fig. 1).

### Presentation of the Process of Impact and Acceptability Analysis Devised by Our Team

Since 2012, based on this conceptual frame of reference, our interdisciplinary research group has been building an analytic framework that takes the form of a reflective process of impact and acceptability analysis, with a view to applying it to a specific case: that of a polymer shoe sole incorporating carbon-nanotube-based sensors for measuring pressure peaks for the purpose of enhancing diabetic care, more precisely by helping to prevent pressure sores.

This process consists of three consecutive moments, as indicated in Table 1 below.

**Table 1:** The three moments of the process of impact and acceptability analysis

Moment 1: Identifying impacts on specific issues
Stage 1: Identifying the technological source that could have an impact on a specific issue
Stage 2: Identifying a specific issue that could be subject to impacts from that source
Stage 3: Determining the real impact of the source on the issue
Moment 2: Assessing impacts based on the values selected
Stage 1: Characterising the impacts on each issue in terms of values
Stage 2: Final assessment judgement regarding positive or negative impact on each issue
Moment 3: Assigning weight to the final assessment judgement in view of reaching a decision
Stage 1: Determining the kind of weighting to apply: Acceptability of risks or comprehensive acceptability of impacts?
Stage 2: Weighting process: (a) according to acceptability of risks; or (b) according to comprehensive acceptability of impacts

Let us now see how this process of comprehensive impact and acceptability analysis may be applied to the pressure sensor using carbon nanotubes and designed for health purposes.

#### Moment 1: Identifying Impacts on Specific Issues

The goal of this first moment is to carry out scientific analyses allowing for: (1) tracing the technological source that could have an impact on a specific issue; and (2) taking account of the context in order to verify that this technological source applies in the case in hand. The point of departure for identifying impacts is the same as that of classical risk analysis, because it is necessary to establish the existence of a relationship between a source and its impact, on the one hand, and the situations in which the impact will arise, on the other hand.

##### Stage 1: Identifying the Technological source that Could Have an Impact on a Specific Issue

In the present case, what is under discussion is a polymer shoe sole that incorporates a sensor with carbon nanotubes (i.e., a use of a nanotechnology). In classical risk analysis, what would be taken into account would be essentially the nanotechnological component, i.e., the carbon nanotubes. The goal would be to determine whether: (i) exposure to carbon nanotubes can have toxic effects on human beings; and if so, what quantities are necessary (dose–response) to produce such effects (Occurrence 2 in the conceptual frame of reference); and (ii) in the case of the rupture of the polymer shoe sole, whether the quantity of liberated nanotubes could produce toxic effects in exposed humans (Occurrence 1 in the conceptual frame of reference).

In a classical analysis, the device would be viewed as a nanomaterial (carbon nanotube) as well as a finished product (shoe sole incorporating carbon nanotubes). In instances where a classical risk analysis is dealing with safety, it is the manufacturing processes for carbon nanotubes and the finished product that are taken into account. The question would be: Are workers being exposed to carbon nanotubes in such a way as to prejudice their health?

In contrast, when we take into consideration the various perspectives on nanotechnologies represented by the social sciences and the humanities, we realise that analyses based in those disciplines advocate also taking into account, for the purposes of impact assessment, the final product’s various uses as well as the impacts of this kind of nanotechnological research on science itself.

The finished product, the polymer shoe sole incorporating carbon nanotubes, is intended for a medical purpose: Because foot sensitivity is reduced by the disease, the shoe sole conveys to caregivers information about the pressure exerted on the sole and thus about the state of health of the foot of the diabetic user, allowing caregivers to adjust care. However, products originally intended for one designated purpose are often used by society for another purpose. In this instance, the signals emitted by the carbon nanotube shoe sole may make it possible to convey patient data to duly authorised caregivers; but these signals could also be used as the equivalent of a global positioning system (GPS) by other individuals in order to monitor patients’ movements from a distance. The latter use could constitute an invasion of privacy. In the eyes of many, the impacts of such secondary uses are just as important in risk assessment as health and safety impacts.

Research on biotechnology and nanotechnology is, as the names reveal, technological research focused on end uses. Producing more accurate sensors by using carbon nanotubes or other nanoparticles is the engine that drives the source and the source’s funding. According to some researchers in the field of critical sociology—for example, Lafontaine (2010)—analysis of the impacts of nanotechnologies is incomplete if it does not take into account the social issues associated with the development of this kind of research.

Table 2 below indicates the diversity of sources associated with nanotechnologies that have been taken into consideration in various opinions, studies, and reports.

**Table 2:** Diversity of sources associated with nanotechnologies

*(I) Products*
(a) Nanomaterials—nanoassembly—nanosystems
These manufactured products are those on a nanometric scale that may manifest specific properties on that scale (e.g., carbon nanotubes)
(b) Intermediate nanoproducts
These result from a series of manufacturing stages at the nanometric scale or the macroscopic scale. Except for potential uses in monitoring and assessing manufacturing processes, the resulting object has no specific use
(c) Finished nanoproduct
These are the devices created using a nanoproduct (a product produced by incorporating nanomaterials, nanoassembly, or nanosytems into a substrate), in line with a general purpose but without aiming at a specific use (e.g. pressure sensor incorporating carbon nanotubes)
(d) End-use nanoproduct
These are products created for specific uses and using finished nanoproducts (e.g., a sole incorporating a pressure sensor based on carbon nanotubes)
*(II) Procedures*
(a) Procedures for nanomaterial manufacture—nanoassembly—nanosystems
The set of technical and industrial procedures put into place to manufacture these products
(b) Procedures for manufacturing intermediate and finished nanoproducts
The set of technical and industrial procedures put into place for manufacturing finished nanoproducts
(c) Procedures for manufacturing end-use nanoproducts
The set of technical and industrial procedures put into place for manufacturing these end-use nanoproducts
*(III) The Process*
This is the technical-scientific process that has framed technological development since the mid-twentieth century. This process consists of the over-determination of economic considerations in the making of choices about scientific research, which orients scientific research towards technological developments that can be rapidly marketed
*(IV) Uses*
All uses other than the one originally intended for an end-use nanoproduct (e.g., a pressure sensor that serves to improve health care for diabetic patients but that also serves to monitor these same individuals’ movements)

The IRGC’s policy brief proposes two “Frames” for risk governance, depending on the generation that nanostructures and nanoproducts belong to (IRGC 2007, 8 and passim). In the first generation, we have passive nanostructures used in metals, polymers, aerosols, colloids, and so on. These are addressed by the traditional risk governance approach. The IRGC is concerned about the later generations of nanostructures, like targeted drugs and biodevices, which will be active. This classification of nanoproducts and nanostructures shows the complexity not only of the nanomaterials involved but also of the materials’ varied uses in the medical, biological, and technological realms (Renn and Rocco 2006). Our framework goes one step further by distinguishing between the various sources of nanotechnological impacts involved in an impact analysis. We can gain a clearer understanding of the complexity of the governance of nanotechnological impacts by distinguishing between sources and identifying their possible impacts on the issues concerned.

##### Stage 2: Identifying a Specific Issue that Could Be Subject to Impacts from that Source

Each of the preceding aspects of the technological source can have a different impact on what we refer to as a specific issue. For example, the fact that the sensor is made of carbon nanotubes raises the question of toxicity (negative impact) to humans who are exposed to it (health issue). If the sensor can result in death, the question of toxicity can be situated in relation to death (life/death issue). It can also be situated in relation to the environment (environmental issue).

If the sensor is looked at from the perspective of its being part of the process of technological development, the question arises of economic impact (economic issue), but so also does that of an impact on the status and development of scientific research (issue of status and development of scientific research).

When the sensor is looked upon as a finished product, it is understood to be intended to provide information as part of an expected and predetermined function, for example, preventing pressure sores in diabetics (health issue). On the other hand, the information obtained could also be used to track the wearer of the soles (freedom of choice issue and privacy issue). Incorporating the sensor into health care could have an institutional impact locally and nationally (issue of cohabitation at the local and national levels), as well as internationally when it comes to the accessibility of these soles in developing countries (issue of cohabitation at the international level). What if the sensor could be incorporated into the patient’s foot? In that event, there would be impacts for cultural representations of the human being (issue of cultural representations of the human being, identity, nature, the person).

In sum, identifying an issue assumes that, whether personally or collectively, we assign significance to that issue in our manner of living individually and as a society.

In various ethical opinion statements, these issues are collectively designated as E^3^LS (ethical, environmental, economic, legal, and social). An E^3^LS perspective dismantles the old EHS/ELS dichotomy. As seen above, the issues in question can be arrayed along the following ten general dimensions:

1. health
2. life/death
3. the environment
4. the economy
5. the status and development of scientific research
6. freedom of choice
7. privacy
8. cohabitation (local—national)
9. cohabitation (international)
10. cultural representations of the human being (identity—nature—the person)

In our framework, we do not distinguish between risks and impacts, because fundamentally, risks are negative impacts on an issue. An EHS risk analysis concentrates exclusively on impacts on environmental, health, and safety issues. But nanotechnology has impacts of other kinds, for example, on the economy. In a traditional risk analysis, the judgement about acceptability is based on a trade-off between risks and economic benefits. The IRGC’s policy brief discussed above recommends that in conducting a risk appraisal, we take into account what the document calls societal concerns (IRGC 2007, 8, 18, 22, 26), including “the social and moral implications of future innovations” (19). A closer look at these societal concerns reveals that they refer to the impacts of nanotechnology on our ways of living in society. The impacts of the use of nanotechnological devices on issues such as privacy, human identity, national security, etc., are not just concerns; they are real impacts of the uses of nanotechnology in our world. This is why in our framework we treat societal concerns as social impacts that must be analysed just as seriously as toxicological impacts.

##### Stage 3: Determining the Real Impact of the Source on the Issue

In conducting a scientific analysis, we must specify on which study we are basing the determination of positive or negative impacts on a specific issue. We must also take into account the concrete situation in which people may be exposed to the technological source. In the case of a specific situation, it’s a question of establishing the probability that exposure to carbon nanotubes will arise. For example, if carbon nanotubes are incorporated into a polymer shoe sole, what is the probability that an individual will be exposed to those carbon nanotubes? And in the worst case scenario, will such exposure be sufficient to generate a negative health impact?

In sum, these three stages that go to make up the first moment in the analytic process come together to result in the identification of what constitutes a problem, in the definition of the object of analysis, or in the bringing out of aspects that raise questions; and they do so with maximum rigour and precision. This initial basis, which is all too often neglected in debates by both experts and members of the public, thus requires that two occurrences be placed in relationship with each other. The first occurrence is identified by scientific and scholarly studies (in the natural sciences and engineering, the health sciences, or the social sciences and the humanities), which make it possible to establish a general relationship between the source and its impact on a specific issue. Some examples: a link between exposure to carbon nanotubes and cancer; a link between technological development and a nation’s economy; a link between cultural transformations and social problems; etc. The second occurrence situates the preceding general occurrence in a particular case: What is the possibility, in the event of the breakage of a shoe sole containing carbon nanotubes, that a person will experience sufficiently high exposure to produce toxic effects or cancer? What is the possibility that nanotechnological development in Quebec will be significant enough to produce an impact on the province’s economy? What is the possibility that the manufacture of electrical batteries using DNA will affect social representations and produce social problems?

Debates around analysis of negative and positive impacts often relate to the bases for these analyses. Thus impacts from potential secondary uses that arouse concern may be dismissed as “groundless fears” (Ginon and De Rochegonde 2008). On the other hand, the critique of risk management exclusively based on known risks has resulted in the precautionary principle. In light of various impact analyses, we differentiate among four kinds of scientific knowledge that support the probability of an impact emerging from a given source (i.e., Occurrence 1), as indicated in Table 3 (Legault et al. 2012).

**Table 3:** The four types of scientific knowledge that support the probability of an impact’s occurrence

(I) Known impact
Empirical knowledge or scientific data about the relationship that makes it possible either to know the probability of the occurrence or to know the relationship only
(II) Probable impact
Empirical or scientific knowledge that makes it possible to establish a hypothesis about the relationship, although aspects of the studies are controversial
(III) Hypothetical impact
Knowledge that exists based on an experiential analogy that makes it possible to formulate a hypothesis about the relationship
(IV) Theoretical impact
No scientific knowledge, so that it is not possible to conclude the relationship does not exist

As is clear, there can be greater or lesser degrees of clarity about a causal relationship between the types of knowledge deployed and the impacts we are seeking to explain, prove, control, indeed predict. Hence the importance within impact analyses of keeping in mind the distinction between scientific uncertainty and scientific ignorance. The term “scientific uncertainty” is reserved, in the social sciences and the humanities and more specifically in applying the precautionary principle, for a situation of scientific controversy about a specific subject (for example, the probable impact of mobile phones on the brain) (AFSSET 2005, 36–37). The term “scientific ignorance” refers to a situation in which we have no scientific knowledge (that is, no reliable studies) on a given subject.

At the close of these three stages that constitute Moment 1 of the analytic process, it becomes possible to better identify the positive and negative impacts that are to be taken into consideration in determining the acceptability of products and their uses in society.

It is at this point that the significance of the second moment of the process, that of assessment, comes into play.

#### Moment 2: Assessing Impacts Based on the Values Selected

In a toxicological risk analysis, studies determine, for example, whether a given degree of exposure to a product could cause, with a given degree of probability, lung cancer (as in the case of exposure to tobacco) or neurotoxic effects (as in the case of the consumption of fish with high mercury levels). All these analyses implicitly presuppose the following assessment: the less an individual is exposed, the less health values are diminished; and inversely, the more an individual is exposed, the more health values are diminished. It is this assessment based on health that will be used in Moment 3 for the purpose of issuing a statement on risk acceptability.

The purpose of Moment 2 is to systematically assess selected positive and negative impacts in impact determination. The assessment consists of a value judgement that, for each positive or negative impact, establishes the level of maximisation or minimisation of the value associated with it.

Risk governance is value laden, and every governance framework refers, implicitly or explicitly, to value judgments that are embedded in the judgement of acceptability. Since risk governance depends on these value judgments, special care is required in defining the status of a value judgment involved in risk appraisal. The IRGC policy brief identifies values as social or national and cultural and classifies risk problems as “simple, complex, uncertain, and ambiguous” (IRGC 2007, 22). Renn and Rocco (2006) address the question of values as discussed in the IRGC policy brief by interpreting this use of the word “ambiguous”. The word ambiguous, they say, has two meanings. The first relates to data that can be interpreted in different ways. The second meaning is normative:

Secondly, it denotes a variability of normative evaluation with respect to the tolerability or acceptability of observed effects on a given value or norm. Many scientific disputes do not refer to differences in methodology, measurement or dose–response functions, but to the question of whether the observed or assumed impacts violate or meet the predefined values. Often it is also contested which values are (will be) actually of issue or are (will be) subjected to discussion and how essential these values are and for which group. (Renn and Rocco 2006, 163–164).

Our framework proposes an analytic way of clarifying the value judgements made regarding the impacts considered, as well as identifying the reasons why a given group considers certain values as essential in an impact appraisal.

##### Stage 1: Characterising the Impacts on Each Issue in Terms of Values

Identifying a specific issue in determining the nature of impacts is not a neutral matter. By definition, it indicates that the specific dimension of our lives that may be subject to a given impact is significant for us, either individually or collectively. In other words, identifying an impact presupposes that this dimension has value, if by “value” we understand something that is the object of a preference, something that is prized, preferred, or desired.

With each issue selected, it is possible to associate a value that we find in it. Generally it’s a question of quality. Why, for example, is the issue about health so important to us? Because we seek to maximise the quality of health for each person. Take the shoe sole containing a sensor that uses carbon nanotubes: if it is intended to improve the health of diabetics, it can be said that it seeks to maximise the quality of health for a diabetic. On the other hand, if a diabetic is exposed to the toxic effects (the impact) of carbon nanotubes, this minimises the quality of that person’s health.

Thus when a group or commission prepares an ethical opinion, its members must establish consensus on the values associated with the general issues, which relate more specifically to the quality of what is being targeted:

1. quality of human health
2. quality of individual life/death
3. quality of the environment
4. quality of economic impacts
5. quality of the development of scientific research
6. quality of individual autonomy (freedom of choice)
7. quality of privacy
8. quality of cohabitation (relations among individuals within a state)
9. quality of cohabitation (international relations)
10. the value of our cultural representations of the human being (identity, nature, the person)

##### Stage 2: Final Assessment Judgement Regarding Positive or Negative Impact on Each Issue

The final assessment judgement consists precisely of indicating the degree of maximisation (optimisation) or minimisation (deterioration) of the value produced by a positive or negative impact. Based strictly on our desires, we all want total quality: total health, total quality in our love relationships, total quality in our economic well-being…. In other words, we all wish for Paradise.

Assessment may appear subjective and arbitrary, but in reality, a logic of assessment exists, and it can be spelled out as follows. Everything is rooted in our desire to grant significance to certain ways of living for ourselves and with others in the most just possible society. The values identified represent these various prioritised ways of living that enable us to assess positive and negative impacts.

For example, in the case of the sensors discussed above, working with the same impact studies, some people will deem that the actual impact minimises quality of health to some degree (a little, somewhat, a good deal, almost fully) while others will deem that the actual impact maximises it to those same varying degrees. The variation among the assessments is to be explained by degree of significance assigned to the least change in the diabetic person’s health. For some people, even a tiny gain is in itself a great improvement in quality of life. On ethics boards, dialogue around each impact assessment makes it possible to reduce subjective gaps.

#### Moment 3: Assigning Weight to the Final Assessment Judgement in View of Reaching a Decision

##### Stage 1: Determining the Kind of Weighting to Apply: Acceptability of Risks or Comprehensive Acceptability of Impacts?

As we have already emphasised, the classical analysis of toxicological impacts focuses exclusively on risk analysis. This approach implicitly assumes that the final judgement about accepting or rejecting a technological product rests exclusively on a refusal to take a risk deemed unacceptable. In other words, no benefits from the product can compensate for loss of life or health. When the analysis is not exclusively reduced to health and safety risks but examines all possible impacts, it becomes necessary to weight not just a risk but the set of all the impact assessments.

The choice between two kinds of weightings and the reasons for the choice are significant, for they relate to differing expectations about the regulation of technological development in our societies. Should the State limit itself to regulating known health and safety risks; or should it assess and weight the set of all impacts associated with the management of technological development?

##### Stage 2: Weighting Process: (a) According to Acceptability of Risks; or (b) According to Comprehensive Acceptability of Impacts

- According to acceptability of risks

Risk analysis focuses exclusively on negative impacts and predicts their probability and intensity. Risk assessment is then conducted according to the issue in question. Whereas toxicological analyses assess impacts based on the value of health quality or danger to life, some philosophical analyses assess the impact that uses will have on human dignity or the human condition.

The final judgement about the unacceptability of a risk rests on the extent of reduction in the assessment’s reference value. To gauge the extent of reduction, the weighting of risks must deploy arguments that make it possible to show that reduction. In the field of toxicological studies, the point of departure consists of scientific standards that establish thresholds for exposure beyond which there is a high probability of disease or death. Here, the weighting rests on a single value, that of health and life; and it is deemed that, up to a certain threshold of exposure, risk is acceptable and after that threshold it is no longer acceptable. From a sociological perspective, the probability that a technology will be accepted de facto by a given population becomes the criterion for deeming whether a risk is acceptable or not. Thus the probability that the pressure sensor will not be rejected by the public in the future leads some to deem it acceptable. In contrast, some finished products, like biological sensors built using DNA as a raw material, could have an impact on representations by blurring the frontiers between the natural and the artificial. For some philosophers, an assessment impact based on the concept of nature in civilisation makes such forms of manipulation unacceptable. Many philosophical arguments have been deployed as bases for deeming technologies acceptable or unacceptable (Patenaude et al. 2011), and this raises a major issue about the role of philosophy in interdisciplinary analysis (Legault et al. 2013).

(b) According to comprehensive acceptability of impacts

Comprehensive impact analysis makes it possible to identify multiple impacts on various issues and assess them in the light of values specific to each issue. An analysis of this kind allows for a better understanding of the complexity of individual choice as well as of social choice. In contrast to risk analysis, which is based on a single essential and preponderant value, the comprehensive impact analysis must take into account the full set of assessments. Weighting the impact assessments can be imaged as a placing of the negative and positive assessments on each pan of an old-fashioned scale. In many settings, cost/benefit analyses conducted exclusively on the basis of economic criteria prevail. In sociology, the weighting would be based on current or future mores as related to the weighting that individuals currently assign or that we can project they will assign in the future in relation to these assessments. In philosophy, the utilitarian approach, based on the greatest happiness of the greatest number, advances the principle of weighting according to the good, whereas the moral argument based on equity requires that the impacts of technologies be weighted in line with the equitable treatment of individuals. In political philosophy, we encounter the argument that it is democratic institutions that de facto determine the final weighting. Finally, in the field of applied ethics, the weighting of various assessments is required to be done by showing how each component considered to be preponderant contributes to the kind of life that constitutes quality of life within society.

## Conclusion

The GMO crisis gave rise to numerous enlightening studies on the inadequacies of the principle of social acceptance embedded in the existing approach to the regulation of technological development. A shift from a free-market development approach to a responsible development one brings into focus the necessity to take into account the responsibility of all the actors involved. “The emerging concept of RRI (responsible research and innovation), however, confers new responsibilities; and not only on scientists but on universities, innovators, businesses, policy-makers and research funders” (Owen et al. 2012). But in order to establish a dialogue between all the actors, including consumers, there must be a shift in our approach to risk governance. Not only the toxicological impacts of nanotechnologies must be considered but also the social impacts of the various uses of nanoproducts. Moreover, such a shift also implies re-examining the role of values and value judgements in the process of ethical assessment and understanding how they operate in decision-making. Finally, it challenges democratic societies to open up to public debate the social choices involved in developing a specific technology.

It is in order to meet the needs of this shift that we developed a single comprehensive framework for the analysis of impacts and acceptability based on interdisciplinary expertise, a systematic approach structured by three moments. These are: Moment 1, Identifying impacts on specific issues; Moment 2, Assessing impacts based on the values selected; and Moment 3, Assigning weight to the final assessment judgement with a view of reaching a decision.

This approach is designed to take into account all the sources associated with nanotechnological development that could have an impact on any of the E^3^LS issues. Moreover, it also aims to open up discussion about the underlying reasons for the existence of a real impact by a source on a specific issue. The same is true of assessments of these impacts and the weighting of the assessments that are generally left implicit in risk analysis. By opening up discussion about what underlies the assessment and weighting processes, we can better understand the arguments on which they are founded.

A framework of this kind for analysing impacts and accessibility enables us to better understand the multiplicity of positions on nanotechnologies and the reasons why these positions seem inaccessible to any form of argumentation. We believe that if we wish to surmount the many antagonisms in debates about nanotechnology, this analytic framework will serve as a tool indispensable to any individual, group, or committee formulating ethical opinions and wishing to take a stand on nanotechnological devices. Similarly, this reflective tool could serve researchers and industrial backers as a way of integrating the dimension of ethical acceptability into the process of development of devices.
